# Frequency and risk factors for tail injuries in UK dogs under primary veterinary care

**DOI:** 10.1002/vetr.6020

**Published:** 2025-10-14

**Authors:** Camilla Pegram, Dan G. O'Neill, Alexandra Edwards, David B. Church, Dave C. Brodbelt

**Affiliations:** ^1^ Pathobiology and Population Sciences Royal Veterinary College Hatfield UK; ^2^ Clinical Science and Services Royal Veterinary College Hatfield UK

## Abstract

**Background:**

Tail injuries in dogs can severely impact welfare and pose clinical challenges. This study aimed to describe the annual incidence and clinical management of tail injury in UK dogs under primary care and identify risk factors.

**Methods:**

A nested case‒control design was used within a cohort of dogs under primary care in the UK in 2019. Clinical records were automatically searched and manually verified to identify incident tail injury cases and extract management data. Multivariable logistic regression was then used to evaluate associations between potential risk factors and tail injury diagnosis.

**Results:**

The analysis included 285 tail injury cases and 285,000 randomly selected controls. The estimated annual incidence risk for tail injury was 0.23%. Increased odds of injury were observed in boxers, English springer spaniels and cocker spaniels; dogs aged less than 2 years; male entire, male neutered and female neutered dogs; working and gundog breeds; and dogs weighing between 20 and 30 kg. Systemic analgesia and antibiosis were prescribed in 45.6% and 32.6% of cases, respectively. Surgical amputation was undertaken in 9.1% of cases.

**Limitations:**

This study relied on retrospective electronic health record data. Only factors consistently recorded in clinical records were available.

**Conclusion:**

Tail injury is relatively uncommon but carries serious welfare risks. Identifying high‐risk groups offers valuable insights for veterinarians, breeders and policymakers.

## INTRODUCTION

Tail injuries in dogs can be challenging for clinicians to manage successfully due to factors such as limited available mobile skin for reconstructive surgery and frequent complications in wound healing.^1^ A naturally functioning tail is important for canine communication, balance and scent marking; therefore, injuries may impair these functions and also pose a significant welfare concern due to associated pain.^2^ Elective tail docking (the partial or complete removal of a dog's tail) was common practice historically in the UK for several dog breeds, for reasons including culture, aesthetics and injury prevention.^3^, ^4^ However, since 2007, tail docking has been banned in England, Wales and Northern Ireland, except when performed by a veterinary surgeon for certain working dogs or for medical purposes. In 2017, the ban on tail docking that had been imposed in 2007 was reversed in Scotland to permit the docking of tails—by up to a third—in spaniels and hunt point retriever (HPR) breeds that are likely to be used for work.^5^

Previous studies have reported the prevalence or incidence risk of tail injuries in dogs in different populations.^6^, ^7^, ^8^ Cameron et al.^8^ estimated a 10‐year prevalence of 0.59% in dogs attending 16 veterinary practices in Scotland, with annual prevalence ranging from 0.11% to 0.32%. Darke et al.^6^ reported an annual prevalence of 0.39%, based on dogs in Edinburgh. Diesel et al.^7^ reported an annual incidence of tail injury of 0.23% in dogs attending 52 practices in Great Britain. However, the incidence risk of tail injuries in dogs has not previously been reported using data from a large, nationally representative sample of veterinary practices across the UK.

Previously reported tail injuries in dogs include lacerations, fractures, dislocations, self‐trauma, neoplasia, unspecified tail tip injuries, blunt trauma, contusions and ‘water tail’.^7^, ^8^, ^9^ Among these, lacerations were the most commonly reported injury, accounting for up to 70% of tail injury cases.^7^

Breed has previously been identified as a risk factor for tail injury, with English springer spaniels, greyhounds, lurchers and whippets reported to be at higher risk than Labrador retrievers and other retrievers.^7^ A study exploring tail injuries in working gundogs and terriers in Scotland identified HPR breeds and spaniels as being at greater risk than other working breeds.^10^ Elective tail docking has been associated with a reduced risk of tail injury.^7^, ^9^, ^10^ It has previously been found that dogs under primary veterinary care in Great Britain with docked tails had 0.03 times the odds of sustaining a tail injury compared to dogs with undocked tails.^7^ Another study focusing on working dogs reported a 20‐fold reduction in the likelihood of tail injury when tails were docked by one‐third or more.^10^ Additionally, dogs kept in kennels (during night, day or both) and a wide tail wag angle have been identified as risk factors for increased tail injury.^7^ While working dog breeds have been reported to have a higher risk of tail injury compared to non‐working breeds.^7^, ^8^ Diesel et al.^7^ did not identify explicit ‘work use’ (i.e., being classified as a working dog rather than a pet dog of a working breed) as a risk factor for tail injury.

Limited data are available regarding the current clinical management of tail injuries in dogs under primary veterinary care. Diesel et al.^7^ reported that 57.7% of injuries were treated conservatively with antibiotics, anti‐inflammatories and dressings, with 30.9% resulting in tail amputation and 11.4% of cases not requiring any specific treatment.

Using anonymised veterinary clinical data from the VetCompass programme,^11^ this study aimed to evaluate the annual incidence of tail injuries in dogs, describe their clinical management and identify associated risk factors. Based on previous evidence,^7^, ^8^ the study hypothesised that gundog and working dog breeds would have greater odds of a tail injury than non‐gundog or working breeds.

## MATERIALS AND METHODS

### Study design and power calculation

VetCompass collates de‐identified electronic health record (EHR) data from primary care veterinary practices in the UK for epidemiological research.^11^ The study population included all available dogs under primary veterinary care at clinics participating in the VetCompass programme during 2019. Dogs under veterinary care were defined as those with at least one EHR (free‐text clinical note, treatment or bodyweight) recorded during 2019. Available data fields included a unique animal identifier along with species, breed, date of birth, sex, neuter status, insurance status and bodyweight, and also clinical information from free‐text clinical notes and treatment information with relevant dates.

The study used a nested case‒control design within the underlying cohort of dogs under veterinary care in 2019. Sample size calculations in Epi Info (CDC) estimated that approximately 222 cases and 222,000 controls would be required to identify if dog breeds classified as gundogs by the Kennel Club had at least 1.5 times the odds of tail injuries compared to other breed groups, assuming that 36% of UK dogs are gundogs (based on 2019 Kennel Club breed registration statistics),^13^ a 1000:1 ratio of control:case, 80% power and 95% confidence (Epi Info 7 CDC, 2019). A 1000:1 control:case ratio was selected to ensure sufficient power to detect differences for less common categories within variables.

### Case finding and definition

The case definition for a tail injury required a final diagnosis of a tail injury recorded in the EHR, including lacerations, fractures, dislocations, contusions, abrasions, punctures, degloving, self‐trauma and unspecified ‘wounds’. Exclusion criteria included neoplasia, unspecified ‘masses’, self‐trauma that resolved after anal gland expression and acute caudal myopathy. Acute caudal myopathy was excluded as it typically does not involve external trauma, is often of uncertain aetiology^14^ and was not consistently described by clinicians as a tail injury.

Only incident cases were included in the study, with these cases defined as dogs with a tail injury first diagnosed between 1 January and 31 December 2019. Candidate cases were identified by applying search terms relevant to the tail injury diagnosis in the clinical notes during 2019 (including tail +: inju*, woun*, lacer*, abra*, brui*, frac*, disloc*, trau*, ‘self‐trauma’). The search findings were merged, and a random subset of candidate cases, randomly presented through the online database using the RAND function in SQL Server,^15^, ^16^ had their clinical notes manually examined in detail to identify whether they met the case definition. Candidate cases that did not meet the case definition or were not manually reviewed within the available time frame were excluded from the risk factor analysis. All non‐candidate dogs from the underlying denominator population were classified as non‐cases, with a random sample of 285,000 non‐candidate dogs selected as controls. The controls were not manually reviewed; thus, misclassification is possible. However, comprehensive search terms were applied, so any such misclassification is expected to be minimal and likely to bias estimates towards the null, if at all. Demographic data for cases and controls were extracted automatically from the VetCompass database, with further data related to clinical management extracted manually from the EHR for the cases.

### Data preparation

Breed information entered by the participating practices was cleaned and mapped to a VetCompass breed list derived and extended from the VeNom Coding breed list.^17^ A breed purity variable categorised dogs of recognisable breeds as ‘purebreeds’, dogs with names derived from two or more purebred breeds as ‘designer crossbreeds' and dogs recorded as mixes of breeds but without a specific name as ‘crossbreeds’. To maintain sufficient power for analysis, a breed variable included all individual breeds with at least five cases of tail injury or over 5000 control dogs of that breed in the overall disorder risk analysis. The remaining dogs were grouped as either ‘purebreed—other’ or ‘crossbreed’. Breeds were also characterised by haircoat (short, medium, long or uncategorised) and skull conformation (dolichocephalic, mesocephalic, brachycephalic or uncategorised) for analysis, based on a previously published categorisation system.^18^ A Kennel Club breed group variable classified breeds recognised by the UK Kennel Club into their respective breed groups (gundog, hound, pastoral, terrier, toy, utility and working) while all other breeds were classified as non‐Kennel Club recognised.^19^ Neuter status was defined by the final available EHR neuter value and was combined with sex to create four categories: female entire, female neutered, male entire and male neutered.

Adult bodyweight was defined as the median of all bodyweight values recorded for each dog after reaching 18 months of age and was categorised as follows: less than 10 kg, 10 to less than 20 kg, 20 to less than 30 kg and 30 kg or more. The age of cases was calculated at the date of tail injury diagnosis. The age of controls was defined as the age (years) on 31 December 2019. Age was categorised as follows: less than 2 years, 2 to less than 4 years, 4 to less than 6 years, 6 to less than 8 years, 8 to less than 10 years, 10 to less than 12 years and 12 years or more. The veterinary group attended was categorised as 1‒5, representing five distinct corporate practice groups involved in the study.^20^ Where information for a study variable was not documented in the EHR, the status was recorded as ‘not recorded’, and this was included as a separate category in the analysis if ‘not recorded’ accounted for more than 10% of the study variable.^21^

### Statistical analysis

Following data checking for internal validity and cleaning in Excel (Microsoft Office Excel 2013, Microsoft Corp.), analyses were conducted using R version 4.3.3 (R Core Team). The annual incidence risk with 95% confidence intervals (CIs) described the probability of a dog having a first diagnosis of tail injury during 2019. Because the sampling design involved verification of a subset of candidate cases, the total number of ‘true’ cases was estimated by applying the observed confirmation proportion from the reviewed sample to the full candidate case set. The incidence risk was calculated using this estimated count as the numerator, with the denominator comprising the total population of dogs under veterinary care in 2019. The CI estimates were calculated in R from standard errors, based on approximation to the binomial distribution.^22^ Continuous variables were assessed graphically for their distribution and summarised using medians, interquartile ranges (IQRs) and ranges if non‐normally distributed. Student's *t*‐test or Mann‒Whitney *U*‐test was used to descriptively compare continuous variables as appropriate.^22^

Binary logistic regression modelling was used to evaluate univariable associations between risk factors (breed, breed purity, coat length, skull conformation, Kennel Club breed group, adult bodyweight, age, sex‒neuter status and veterinary group) and tail injury diagnosis. A risk factor has been defined as a biological condition, substance or behaviour that has an association with, but has not been proven to cause, an event or disease.^23^ Risk factors with liberal associations in univariable modelling (*p* < 0.2) were taken forward for multivariable evaluation. Model development used manual backwards stepwise elimination. All eliminated factors were re‐evaluated for confounding effects within the provisional final model using the change‐in‐estimate approach, such that a change in the odds ratio (OR) for a primary exposure variable of more than 10% was considered to represent important confounding.^24^ This approach was not intended to adjust for confounding in the context of one specific exposure‒outcome relationship, but rather to report adjusted ORs for each variable of interest, conditional on the others included in the model. If breed, breed purity, coat length, skull conformation, Kennel Club breed group and adult bodyweight (a defining characteristic of individual breeds and therefore highly biologically correlated with breed) were significant at the univariable stage, these variables were excluded from the initial breed multivariable modelling. Instead, these variables, in turn, individually replaced the breed variable in the main final model to evaluate the effects after taking account of the other variables.^19^ Biologically relevant pairwise interactions between final model variables were assessed using the likelihood ratio test with a cut‐off of a *p*‐value of less than 0.05.^25^ The veterinary group attended was evaluated as a fixed effect. The area under the receiver operating characteristic (ROC) curve and the Hosmer‒Lemeshow test were used to evaluate the quality of the model fit.^24^ Statistical significance was set at the 5% level. Figures were created in R statistical software (R version 4.3.3) using the ‘forestplot’ package.^26^

## RESULTS

The study population included 2,250,741 dogs under primary veterinary care at UK practices participating in the VetCompass programme during 2019. Tail injury search terms yielded 54,800 candidate cases (2.4% of dogs). Manual checking of a random sample of 3006 candidate cases (5.5% of candidates) identified 285 confirmed incident tail injury cases during 2019 (9.5% of the checked candidates). After accounting for the subsampling protocol, the estimated annual incidence risk for tail injury in dogs was 0.23% (95% CI: 0.21–0.26).

Descriptive analysis included 285 tail injury cases and 285,000 randomly sampled controls (Table 1). The median age of cases (4.0 years, IQR 1.9–7.8, range: 0.2–15.9) was younger than the median age of controls (5.2 years, IQR 2.2–8.9, range: 0.0–25.0) (*p* = 0.002). The median bodyweight of cases (17.6 kg, IQR 9.3–27.1, range: 1.1–88.0) was higher than the median bodyweight of controls (13.6 kg, IQR 8.4–24.4, range: 1.6–97.5) (*p* = 0.003). The most common breeds among cases were crossbreeds (24.2%; *n* = 69), Labrador retrievers (8.4%; *n* = 24), cocker spaniels (7.7%; *n* = 22), English springer spaniels (3.9%; *n* = 11) and Staffordshire bull terriers (3.5%; *n* = 10). The most common breeds among controls were crossbreeds (23.7%; *n* = 67,577), Labrador retrievers (6.9%; *n* = 19,613), Jack Russell terriers (4.5%; *n* = 12,947), cocker spaniels (4.3%; *n* = 12,224) and Staffordshire bull terriers (4.2%; *n* = 11,829) (Table 1).

**TABLE 1:** Tail injury case count (% of cases) and control count (% of controls) for categorical variables recorded in dogs attending primary care veterinary practices participating in the VetCompass programme in the UK (cases = 285, controls = 285,000).

Variable	Category	Case no. (%)	Control no. (%)
Breed	Crossbreed	69 (24.2)	67,577 (23.7)
	Purebreed—other	89 (31.2)	92,954 (32.6)
	Labrador retriever	24 (8.4)	19,613 (6.9)
	Cocker spaniel	22 (7.7)	12,224 (4.3)
	English springer spaniel	11 (3.9)	5056 (1.8)
	Staffordshire bull terrier	10 (3.5)	11,829 (4.2)
	Cockapoo	8 (2.8)	9168 (3.2)
	German shepherd dog	8 (2.8)	6000 (2.1)
	Jack Russell terrier	8 (2.8)	12,947 (4.5)
	Boxer	7 (2.5)	2146 (0.8)
	Shih‐tzu	7 (2.5)	8561 (3.0)
	Bichon frise	5 (1.8)	3135 (1.1)
	Greyhound	5 (1.8)	1562 (0.5)
	West Highland white terrier	5 (1.8)	4452 (1.6)
	Yorkshire terrier	3 (1.1)	6770 (2.4)
	Border collie	2 (0.7)	7261 (2.5)
	French bulldog	1 (0.4)	8483 (3.0)
Breed purity	Crossbreed	69 (24.2)	67,577 (23.7)
	Purebreed	197 (69.1)	196,934 (69.1)
	Designer crossbreed	19 (6.7)	18,753 (6.6)
	Not recorded	0 (0.0)	1736 (0.6)
Kennel Club breed group	Not Kennel Club recognised	93 (32.6)	91,418 (32.1)
	Gundog	80 (28.1)	47,107 (16.5)
	Hound	19 (6.7)	12,415 (4.4)
	Pastoral	11 (3.9)	16,105 (5.7)
	Terrier	28 (9.8)	36,943 (13.0)
	Toy	15 (5.3)	35,715 (12.5)
	Utility	20 (7.0)	33,842 (11.9)
	Working	19 (6.7)	9719 (3.4)
	Not recorded	0 (0.0)	1736 (0.6)
Coat length	Short	100 (35.1)	101,410 (35.6)
	Medium	73 (25.6)	60,135 (21.1)
	Long	21 (7.4)	23,838 (8.4)
	Uncategorised	91 (31.9)	97,881 (34.3)
	Not recorded	0 (0.0)	1736 (0.6)
Skull conformation	Mesocephalic	133 (46.7)	122,608 (43.0)
	Brachycephalic	32 (11.2)	50,620 (17.8)
	Dolichocephalic	33 (11.6)	23,706 (8.3)
	Uncategorised	87 (30.5)	86,330 (30.3)
	Not recorded	0 (0.0)	1736 (0.6)
Bodyweight (kg)	<10	67 (23.5)	69,035 (24.2)
	10 to <20	73 (25.6)	59,513 (20.9)
	20 to <30	59 (20.7)	37,702 (13.2)
	≥30	39 (13.7)	28,154 (9.9)
	Not recorded	47 (16.5)	90,596 (31.8)
Age (years)	<2	77 (27.0)	64,004 (22.5)
	2 to <4	66 (23.2)	50,510 (17.7)
	4 to <6	44 (15.4)	43,708 (15.3)
	6 to <8	30 (10.5)	37,695 (13.2)
	8 to <10	25 (8.8)	32,435 (11.4)
	10 to <12	24 (8.4)	25,517 (9.0)
	≥12	18 (6.3)	28,908 (10.1)
	Not recorded	1 (0.4)	2223 (0.8)
Sex‒neuter status	Female entire	31 (10.9)	75,400 (26.5)
	Female neutered	87 (30.5)	60,244 (21.1)
	Male entire	75 (26.3)	84,293 (29.6)
	Male neutered	92 (32.3)	62,594 (22.0)
	Not recorded	0 (0.0)	2469 (0.9)
Veterinary group	1	97 (34.0)	98,978 (34.7)
	2	78 (27.4)	76,408 (26.8)
	3	5 (1.8)	4516 (1.6)
	4	47 (16.5)	48,840 (17.1)
	5	58 (20.4)	56,258 (19.7)

**FIGURE 1:**
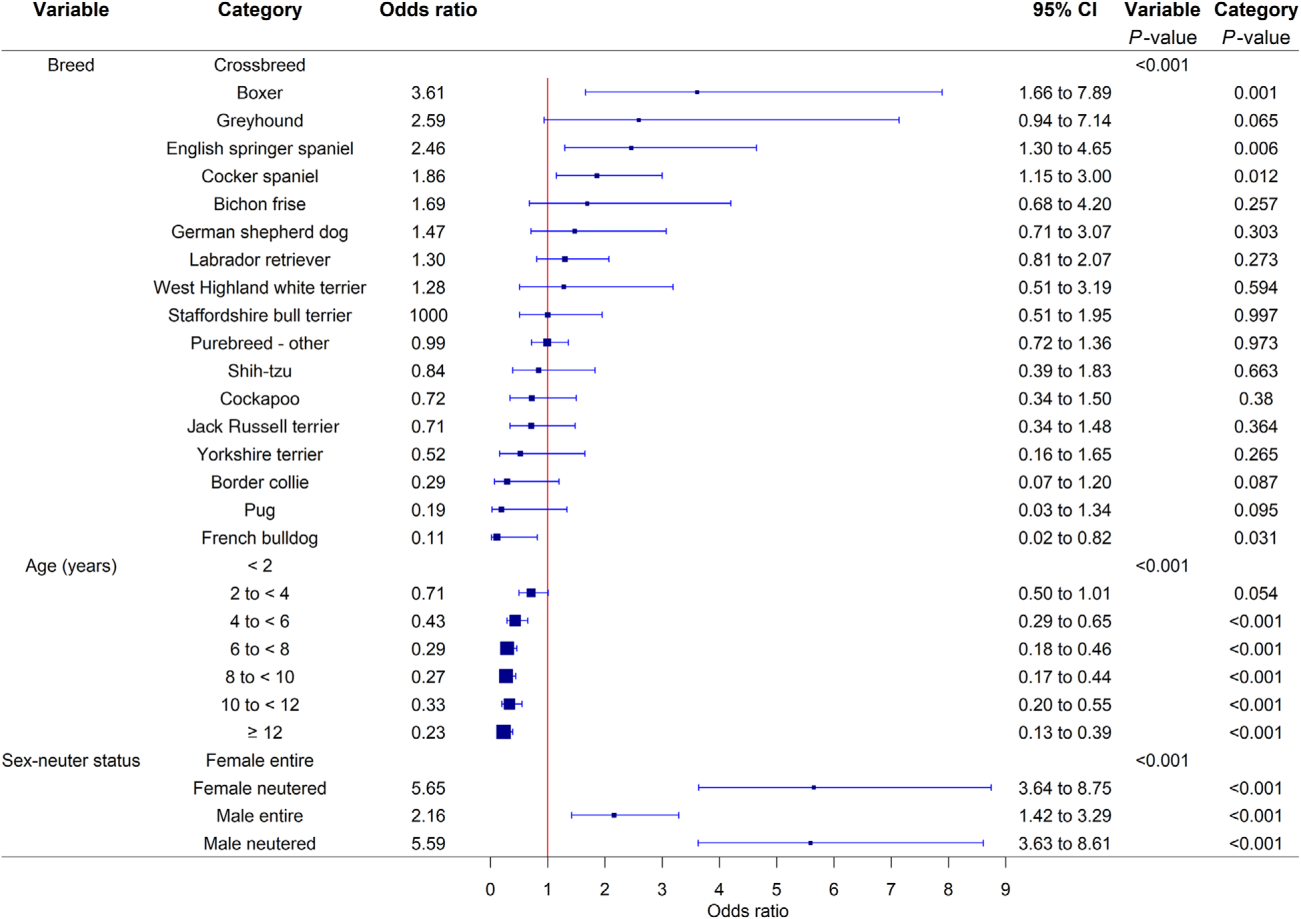
Forest plot of the multivariable logistic regression odds ratios with corresponding 95% confidence intervals (CIs) for demographic risk factors for a tail injury in dogs under primary veterinary care in the UK (cases = 285, controls = 285,000). Categories without an odds ratio were the baseline.

Breed, age, sex‒neuter status, bodyweight, Kennel Club breed group and skull conformation were liberally (*p* < 0.2) associated with tail injury diagnosis in univariable logistic regression modelling, while breed purity and coat length were not. Following evaluation using multivariable logistic regression, the final breed model comprised three factors: breed, age and sex‒neuter status (with the addition of veterinary group as a fixed effect to account for confounding) (Figure 1). No biologically relevant interactions were identified. After accounting for the effects of the other variables evaluated, three breeds showed increased odds of tail injury compared with crossbreed dogs. The breeds with the highest odds were the boxer (OR 3.61, 95% CI: 1.66‒7.89, *p* = 0.001), English springer spaniel (OR 2.46, 95% CI: 1.30‒4.65, *p* = 0.006) and cocker spaniel (OR 1.86, 95% CI: 1.15‒3.00, *p* = 0.012). The French bulldog had reduced odds of tail injury compared with crossbreeds (OR 0.11, 95% CI: 0.02‒0.82, *p* = 0.031). Increasing age was associated with decreasing odds of tail injury, with dogs aged 12 years or more having the lowest odds of tail injury when compared with dogs aged less than 2 years (OR 0.23, 95% CI: 0.13‒0.39, *p* < 0.001). Neutered females (OR 5.65, 95% CI: 3.64‒8.75, *p* < 0.001), neutered males (OR 5.59, 95% CI: 3.63‒8.61, *p* < 0.001) and entire males (OR 2.16, 95% CI: 1.42‒3.29, *p* < 0.001) had increased odds compared with entire females (Figure 1). The Hosmer‒Lemeshow test indicated no evidence of poor model fit (*p* = 0.259), and the area under the ROC curve (*p* = 0.690) indicated acceptable ability to differentiate cases and controls.

**FIGURE 2:**
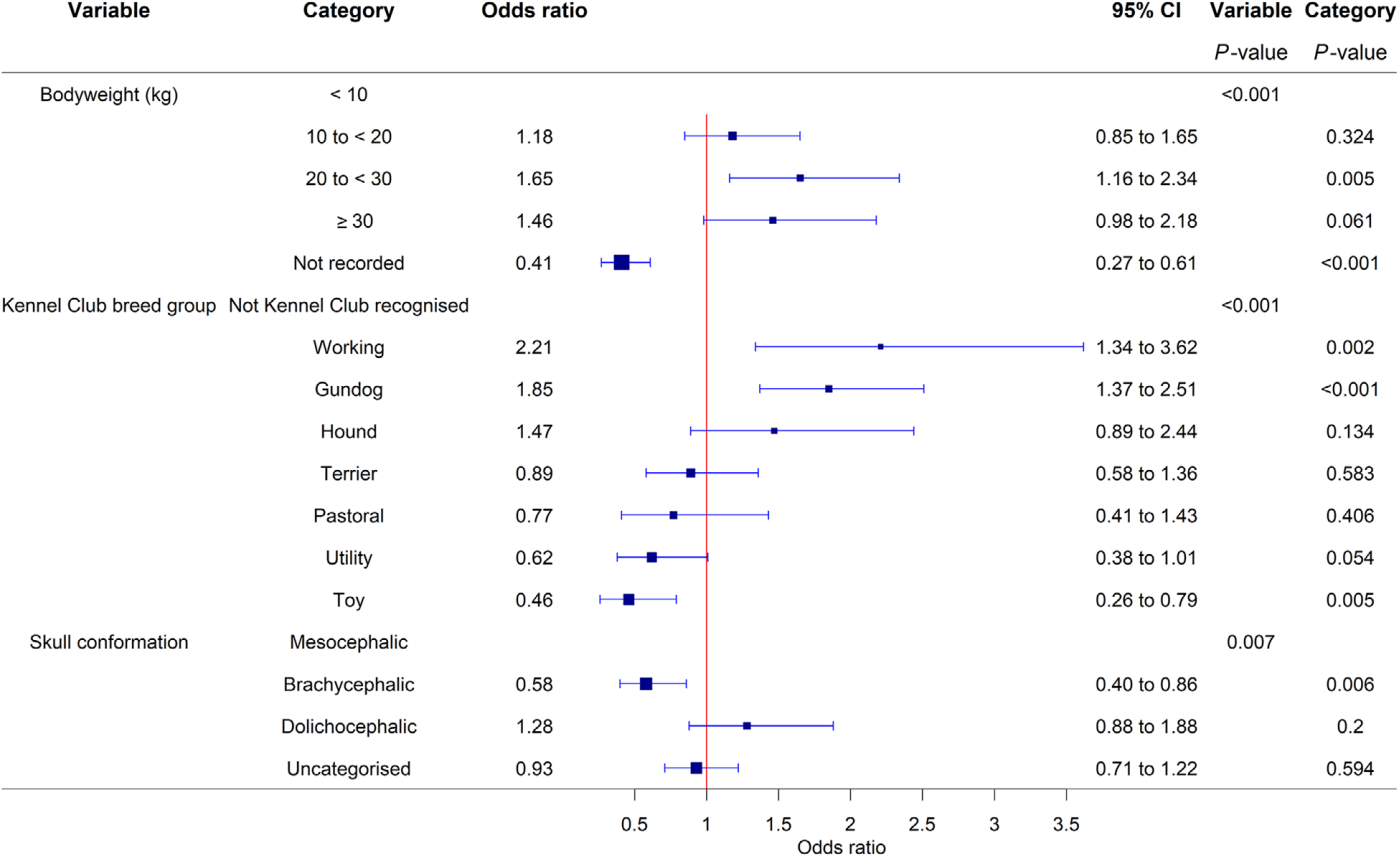
Forest plot of the multivariable logistic regression odds ratios with corresponding 95% confidence intervals (CIs) for adult bodyweight, Kennel Club breed group and skull conformation as risk factors for a tail injury in dogs attending primary care veterinary practices in the UK. These variables individually replaced the breed variable in the original multivariable logistic regression modelling (cases = 285, controls = 285,000). Categories without an odds ratio were the baseline.

Additionally, bodyweight, Kennel Club breed group and skull conformation were significant risk factors when replacing the breed variable in the final breed model. Dogs weighing between 20 and 30 kg had significantly increased odds of tail injury compared with dogs weighing less than 10 kg (OR 1.65, 95% CI: 1.16‐2.34, *p* = 0.005). Compared to breeds that were not recognised by the Kennel Club, working (OR 2.21, 95% CI: 1.34‒3.62, *p* = 0.002) and gundog (OR 1.85, 95% CI: 1.37‒2.51, *p* < 0.001) groups had higher odds of tail injury, while the toy group had reduced odds (OR 0.46, 95% CI: 0.26‒0.79, *p* = 0.005). Brachycephalic breeds had reduced odds of tail injury when compared with mesocephalic breeds (OR 0.58, 95% CI: 0.40‒0.86, *p* = 0.006) (Figure 2).

The most common non‐surgical treatments used to manage tail injury in the 285 cases were systemic analgesia (*n* = 130, 45.6%), systemic antibiosis (*n* = 93, 32.6%), clipping and cleaning of the injury site (*n* = 39, 13.7%), tail bandaging (*n* = 37, 13.0%) and systemic glucocorticoids (*n* = 14, 4.9%). Surgical amputation was undertaken in 26 (9.1%) of cases, while one (0.4%) case was euthanased due to the tail injury. Sixty dogs (21.1%) received no treatment.

## DISCUSSION

This is the largest study to date utilising veterinary EHR data from across the UK to explore the annual incidence and clinical management of tail injury in dogs under primary veterinary care and identify associated risk factors. The reported annual incidence risk was relatively low, at 0.23%, which is in line with Diesel et al.^7^ Boxers, English springer spaniels, cocker spaniels, dogs aged less than 2 years, male entire dogs, male neutered dogs, female neutered dogs, working dog breeds, gundog breeds and dogs weighing between 20 and 30 kg all showed increased odds of tail injury. Systemic analgesia (prescribed in 45.6% of cases) and antibiosis (32.6%) were the mainstay of non‐surgical treatment. Surgical amputation was undertaken in 9.1% of cases. The analyses provide a thorough examination of associative factors and may inform the development of prognostic models. However, these associations should not be interpreted as causal effects, as some variables may act as mediators or be influenced by unmeasured confounders.^27^

The annual incidence risk of tail injury in the current study (0.23%) mirrors the reported estimate by Diesel et al.^7^ in Great Britain. This finding reinforces that tail injury is relatively uncommon, and a stable annual incidence risk from 2009 to 2019 suggests that the overall risk of tail injury in the UK dog population has remained consistent over the past decade. In the Diesel et al.^7^ study, 15.9% of dogs had docked tails, as reported by owners responding to a questionnaire. Given that the UK's tail docking ban was introduced in 2007, a lesser proportion of dogs likely had docked tails in the current study population. The stability in incidence and overall low incidence risk identified in the current study do not provide strong evidence to support reconsideration of current docking legislation. However, this interpretation is speculative, given that tail docking data were not available in the current study. Additionally, this apparent stability may also reflect shifts in breed ownership patterns, including the growing popularity of designer crossbreeds and extreme breeds,^18^ which are at reduced risk of tail injury.

In the current study, breed‐specific risks were a prominent finding. Boxers (OR 3.61), English springer spaniels (OR 2.46) and cocker spaniels (OR 1.86) were identified as being at an increased odds of tail injury compared with crossbreeds. The elevated odds observed in English springer spaniels and cocker spaniels are consistent with previous findings.^7^, ^8^, ^9^, ^10^ However, the ORs in the current study are lower than those reported by Diesel et al.,^7^ in which ORs of 5.97 and 4.75 were reported for English springer spaniels and cocker spaniels, respectively, when compared with Labradors and other retrievers. This may reflect differences in study design and populations, but could also indicate a decline in the use of these breeds for working purposes, thus reducing exposure to hazardous activities. Boxers have not been previously reported as a high‐risk breed. This discrepancy may be attributed to differences in study populations or variations in breed ownership trends over time, potentially influencing the overall risk profile observed in primary care veterinary settings. It could also be that a greater proportion of boxers in earlier studies had docked tails, thereby reducing their previous risk of tail injury.

In contrast, French bulldogs were identified as having decreased odds of tail injury (OR 0.11) compared to crossbreeds in the current study. Additionally, brachycephalic breeds overall had reduced odds of tail injury (OR 0.58) compared with mesocephalic breeds. This reduced risk in French bulldogs, as well as brachycephalic breeds overall, is likely due to their short or ‘screw‐tails’, which provide less surface area for trauma. However, despite the lower risk of tail injury, screw‐tails are associated with vertebral malformations and other health concerns, making them an undesirable trait from a welfare perspective.^28^

When analysed by Kennel Club group, working dog breeds (OR 2.21) and gundogs (OR 1.85) had greater odds of tail injury compared with non‐Kennel Club‐recognised dogs in the current study. Although previous studies have not explicitly grouped breeds according to Kennel Club classification, both working breeds and gundogs have been identified as high risk for tail injury.^7^, ^8^, ^9^, ^10^ This increased risk at a group level may not apply to all breeds in these groups but may instead be driven by specific high‐risk breeds within these groups, namely, boxers, English springer spaniels and cocker spaniels, which were identified as having a significantly higher risk of tail injury in the current study.

Dogs actively used for work are proposed as particularly susceptible to injury due to the physical demands of their roles.^8^, ^10^ While most dogs in the UK today are kept as companions rather than for their traditional working purposes,^29^ inherent breed traits may be linked to injury risk. Working breeds and gundogs are often described as bold, active, playful and highly curious/fearless,^29^, ^30^ characteristics that may predispose them to tail injuries. However, activity type and level of work were not assessed in the current study; therefore, any association between these factors and a tail injury cannot be specifically evaluated.

Toy breeds were at reduced risk of tail injury, which may be attributed to several factors, including their smaller body size, shorter or more finely structured tails, lower activity levels, owner management and temperament. Dogs weighing between 20 and 30 kg were at greater odds of tail injury compared with dogs weighing less than 10 kg when bodyweight replaced breed in the model. Given that many working and gundog breeds fall within this weight range, the observed bodyweight‐associated risk is likely reflective of breed‐specific predisposition rather than bodyweight alone.

Sex‒neuter status was identified as a risk factor in the current study, with female neutered (OR 5.65), male entire (OR 2.16) and male neutered (OR 5.59) dogs at increased odds of tail injury compared with female entire dogs. A previous study did not find a significant association between sex and tail injury, although neuter status was not specifically investigated as a risk factor.^7^ Male dogs have been described as bolder, more aggressive and more exploratory than females,^31^, ^32^ traits that could increase their risk of tail injury. Neutered females and neutered males exhibited a similarly elevated risk of tail injury. The effects of neutering on behaviour are complex,^33^ but behavioural changes following neutering could be associated with injury risk. Additionally, some dogs may not engage in certain behaviours (e.g., working activities) until after they have reached adulthood or been neutered, which may be linked to the likelihood of sustaining injuries later in life. However, it is important to note that neuter status was recorded based on the date of the final EHR, meaning that the timing of neutering relative to the tail injury was not definitively determined for the current study. Therefore, results related to neutering should be interpreted with caution.

The median age at tail injury presentation in the current study was 4.0 years, slightly lower than the value of 5.7 years reported in a previous case‒control study based on primary care data in Great Britain.^7^ Increasing age was associated with decreasing odds of tail injury, with dogs aged 12 years or more having the lowest odds (OR 0.23) of tail injury when compared with dogs aged less than 2 years. While age has not previously been identified as a risk factor for tail injury, it has also not been explicitly modelled as such in prior studies.^7^ Younger dogs tend to be more active, energetic and engaged in vigorous play or exploratory behaviours, which may increase their likelihood of tail trauma.^34^ Additionally, younger dogs may be more likely to participate in working roles, which could contribute to an elevated risk of injury.^8^ Furthermore, the older age group in this study may include dogs born before the 2007 tail docking ban, meaning that a greater proportion of these dogs could have docked tails, reducing their risk of tail injury.

Clinical management of tail injuries was explored and, in line with a previous study,^7^ systemic analgesia and antibiosis were the most frequently used non‐surgical treatments for managing tail injuries. Although the extent of injury for each individual dog was not extracted from EHRs in the current study, the antibiotic prescription rate of 32.6% in the current study could be considered relatively high. Given that antibiotics are not indicated for acute, superficial traumatic wounds,^35^ this finding suggests inconsistent adherence to published guidelines on antimicrobial usage, which further studies could help to clarify. A smaller proportion of dogs in the current study had a tail amputation (9.1%) compared to a previous UK primary care‐based study (30.9%).^7^ This could reflect changes in management strategies but may also be due to differences in case definitions and study design.

The limitations of this study align with those of previous VetCompass publications that utilised similar methods and primarily relied on retrospective analysis of EHR data.^36^ Additional factors, such as tail docking, dog behaviour and lifestyle, may also be associated with tail injury, but information on these factors was not consistently recorded in the clinical records and therefore was not available for the current analyses. Consequently, some dogs in the study population may have had docked tails, potentially influencing the identified risk factors. Future prospective studies might evaluate the association of additional factors, although prospectively collected primary care data may be a more optimal data source for such studies. Additionally, the study likely underrepresents more minor tail injuries, particularly in working dogs, where veterinary care may not have been sought. Nonetheless, the current findings are representative of dogs managed in primary care settings, and thus provide valuable insights into clinically recognised cases of tail injury.

## CONCLUSIONS

The overall annual incidence risk of tail injury in this study (0.23% per year) is consistent with earlier UK estimates, suggesting that the risk of tail injury has not increased substantially in the UK dog population over the past decade. Five risk factors were associated with a tail injury: breed, Kennel Club breed group, bodyweight, age and sex‒neuter status. Boxer, English springer spaniel and cocker spaniel breeds were predisposed to tail injury compared with crossbreeds. Additionally, working dog breeds, gundogs, dogs weighing between 20 and 30 kg, dogs aged less than 2 years, male entire dogs, male neutered dogs and female neutered dogs were at greater risk of tail injury. These findings provide valuable insights for veterinarians, breeders and policymakers by highlighting at‐risk groups.

## AUTHOR CONTRIBUTIONS

Dan G. O'Neill, Dave C. Brodbelt and David B. Church were responsible for the acquisition of the clinical data used in the study. Camilla Pegram, Dave C. Brodbelt and Dan G. O'Neill were responsible for the collation of the study data. Camilla Pegram, Dave C. Brodbelt, Dan G. O'Neill and Alexandra Edwards were responsible for the conception and design of the study. Alexandra Edwards and Camilla Pegram were responsible for the extraction of data. Camilla Pegram carried out the data preparation and analysis. Camilla Pegram, Dan G. O'Neill and Dave C. Brodbelt were mainly responsible for drafting the manuscript. Camilla Pegram, Dan G. O'Neill, Alexandra Edwards, David B. Church and Dave C. Brodbelt were involved in interpreting the results, revised the manuscript, read and approved the manuscript, gave final approval of the version to be published and agreed to be accountable for all aspects of the accuracy and integrity of the work.

## CONFLICT OF INTEREST STATEMENT

None of the authors has any financial or personal relationships that could inappropriately influence or bias the content of the paper.

## ETHICS STATEMENT

Ethics approval was obtained from the RVC Social Sciences Ethical Review Board (reference number: SR2024‐01632811).

## Data Availability

The data that support the findings of this study are openly available in figshare at https://doi.org/10.6084/m9.figshare.29184227.v1.
